# Rapid identification of bacteria utilizing amplified dielectrophoretic force-assisted nanoparticle-induced surface-enhanced Raman spectroscopy

**DOI:** 10.1186/1556-276X-9-324

**Published:** 2014-06-27

**Authors:** I-Fang Cheng, Tzu-Ying Chen, Rong-Ji Lu, Hung-Wei Wu

**Affiliations:** 1 National Nano Device Laboratories, National Applied Research Laboratories, Tainan 74147, Taiwan; 2 Department of Computer and Communication, Kun Shan University, Tainan 71003, Taiwan

**Keywords:** Dielectrophoresis, Microparticle assembly, Surface-enhanced Raman spectroscopy

## Abstract

Dielectrophoresis (DEP) has been widely used to manipulate, separate, and concentrate microscale particles. Unfortunately, DEP force is difficult to be used in regard to the manipulation of nanoscale molecules/particles. For manipulation of 50- to 100-nm particles, the electrical field strength must be higher than 3 × 10^6^ V/m, and with a low applied voltage of 10 V_p-p_, the electrode gap needs to be reduced to submicrons. Our research consists of a novel and simple approach, using a several tens micrometers scale electrode (low cost and easy to fabricate) to generate a dielectrophoretic microparticle assembly to form nanogaps with a locally amplified alternating current (AC) electric field gradient, which is used to rapidly trap nanocolloids. The results show that the amplified DEP force could effectively trap 20-nm colloids in the nanogaps between the 5-μm particle aggregates. The concentration factor at the local detection region was shown to be approximately 5 orders of magnitude higher than the bulk solution. This approach was also successfully used in bead-based surface-enhanced Raman spectroscopy (SERS) for the rapid identification of bacteria from diluted blood.

## Background

Conventional bacteria identification in the hospital typically requires several days for blood culture, bacteria plate culture, and Enterotube analysis [1]. This extensive detection time could lead to rises in death rates and increased drug resistance. Over the past decade, DNA-based detection assays such as DNA microarrays and DNA hybridization to identify bacteria have become popular [2,3]. Antibody-based immunoassays such as the enzyme-linked immunosorbent assay (ELISA) and the Western blot have also been used for detection of microorganisms based on the antibody-antigen interaction [4]. Both DNA-based methods require cell lysing, DNA extraction, DNA amplification, hybridization, and reporter labeling, and antibody-based immunoassays require several complicated steps and long, time-consuming professional operations and are costly because they need elaborate fluorescent/enzyme tagging and sophisticated optical instruments to achieve detection and identification of microorganisms within 12 h [5]. Microfluidic technologies have been popularly employed to reduce the reaction time, required cost, and sample/reagent consumption related to DNA/molecule/bacteria detection due to their miniaturization and high surface area to volume ratio [6,7]. Bead-based assays have the advantage in regard to high collision rate/probability to accelerate DNA-DNA docking and antibody-antigen reactions, and they have been widely used in DNA hybridization and immunoreactions within microfluidic chips [8,9].

Raman spectroscopy is a direct detection platform without complicated sample preparations used for rapid analysis of chemical and biological components based on the measurement of scattered light from the vibration energy levels of chemical bonds following excitation [10]. Unfortunately, Raman signals obtained from biological samples are usually very weak, especially in the case of dilute samples [11]. The use of metallic nanoparticles (NPs) attached on the surface of cells, which is a well-known surface-enhanced Raman spectroscopy (SERS) technique, can generate a higher intensity and more distinguishable Raman signal [12,13]. The generation of coffee-ring effect via droplet evaporation is typically used for the purpose of forming NP-bacteria aggregations naturally [14,15]. Unfortunately, the uniformity of NP-bacteria aggregates is quite random and produces undesirable variations in the spectra [16]. Antibody-conjugated silver NPs (AgNPs) have been used to adsorb on bacteria to produce NP-bacteria aggregation in order to more effectively induce the SERS effect [17,18]. However, these expensive antibodies result in additional costs, and the complicated operations and hours required for antibody modification processes limit the advantages of this method. Antibody-conjugated NP SERS detection also has been shown to produce an additional molecular signal involved in the measured spectrum. For bacteria detection, the SERS effect could only occur at the hot junction of the roughened substrate and the bacterial surface [19]. It is difficult to get an enhanced signal with a low variation due to the fact that the laser light must be focused on the hot junction. In addition, the impurity involved in detecting targets in real blood samples and the low signal to noise ratio associated with bio-objects limits the advantages of SERS technology.

Alternating current (AC) dielectrophoresis (DEP) is the electric field-induced motion of objects via dielectric polarization under nonuniform electric fields. DEP has been widely used for biotechnology applications in micro/nanoscale environments, and it offers a number of potential advantages over conventional methods for cell/bacteria manipulation, separation, and concentration [20,21]. DEP is a flexible tool providing an opportunity to manipulate heterogeneous particles simultaneously. Therefore, the NPs and bacteria could be concentrated to form an NP-bacteria aggregate that serves as a detecting slug for enhancing the Raman spectrum of bacteria. Unfortunately, the DEP force is expressed as a cube function with the particle size (*F*_DEP_ *~ r*^3^); therefore, it is difficult to use DEP force to manipulate nanoscale objects (*r* < 100 nm), such as proteins, viruses, and NPs [22,23].

The platform presented in our work uses a novel concept involving a dielectrophoretic microparticle assembly designed to locally amplify an electric field, and thus, NPs can be manipulated to the surface of microparticles/bacteria in order to conduct an SERS analysis of the bacteria. A simple quadruple electrode with a circular metallic shield at the detection area was designed for separation and concentration of bacteria in the diluted blood and online SERS measurement of the concentrated bacteria, respectively. The bacteria and blood cells (BC) could also be separated based on their different DEP behaviors that depend on their dielectric properties under a specific AC electric field frequency. The challenge of previous works for Raman detection of cells/bacteria/viruses could be addressed through a harmonic combination of the DEP selective tapping of the bacteria from a bacteria-BC mixture and the amplified DEP force-assisted NP-bacteria aggregation used for SERS identification of bacteria.

## Methods

### Chip design, fabrication, and operations

The quadruple electrode array (QEA), compared with other configurations such as interdigitated and castellated electrodes, is easily used for detecting samples in different droplets in order to achieve multiple detections [24]. In quadruple electrodes, the target bacteria can be concentrated at one spot using a negative DEP force to improve detection efficiency even if the bacterial concentration is low. A circular metallic shield was also patterned in the middle region between the quadruple electrodes to reduce the fluorescence noise that could be generated by the laser light penetration of the glass substrate. A 200/35-nm Au/Ti layer was deposited on the glass slides (76 mm × 26 mm and 1 mm thick) using an electro-beam evaporator (JST-10 F, JEOL Ltd., Akishima-shi, Japan). A positive photoresist (AZ 5214, MicroChemicals, Ulm, Germany) was spin-coated on the deposited metal layer, and standard photolithography techniques were employed to determine the designed geometries on the metal layer. After photolithography, wet metal etching was used for microelectrode patterning, and the photoresist was then removed using acetone to complete the microelectrode fabrication.

The bacteria/BC/bacteria-BC suspension sample was placed on top of a quadruple electrode in droplet form, and the motion of the cells was observed under an applied AC field. The DEP behaviors were first characterized by varying the AC frequencies from 100 kHz to 1.2 MHz at a fixed voltage of 15 V_p-p_ to map the DEP properties. The trapping location of bacteria on the electrode edge or in the middle region between the electrodes indicated whether the bacteria exhibited positive or negative DEP at that applied frequency.

### Sample preparation

Five-micrometer latex particles (Sigma-Aldrich, St. Louis, MO, USA) were used to form the nanopores via a dielectrophoretic microparticle assembly. Fluorescent latex particles (Sigma-Aldrich, St. Louis, MO, USA) with a diameter of 20 nm were used for the purpose of observing the nanoDEP mechanism. Five-micrometer latex particles (without fluorescence) and 20-nm fluorescent particles suspended in deionized water (DI) water at concentrations of 5 × 10^6^ and 1 × 10^8^ particles/ml, respectively, were used for validation of the nanoDEP mechanism of the simple chip. *Staphylococcus aureus* (BCRC 14957, Gram positive) and *Pseudomonas aeruginosa* (ATCC 27853, Gram negative) were cultured on tryptic soy agar (TSA) at 35°C. An isotonic solution, a 300-mM sucrose solution with a low conductivity (approximately 2 μS/cm), was used to adjust the conductivity of the experimental buffer solution. To study the separation and detection of the bacteria from the blood cells, a 1× phosphate-buffered saline (PBS) buffer diluted with the 300-mM sucrose solution in a 1:15 ratio was used for the experimental buffer with a final conductivity of 1 mS/cm, owing to the fact that blood cells are highly sensitive to the osmotic pressure of a solution. Bacteria suspended in the isotonic buffer solution with a concentration of 1 × 10^7^ colony-forming units (CFU)/ml and human blood cells were spiked into the prepared bacteria solution in a ratio of 1:400, giving a final blood cell concentration of 10^7^ cells/ml. Silver nanoparticles with a diameter of 40 ± 4 nm (purchased from Sigma-Aldrich, St. Louis, MO, USA) were spiked into the bacteria-BC sample for SERS detection.

### Experimental system

For the purpose of driving DEP forces, a multi-output function generator (FLUKE 284, FLUKE Calibration, Everett, WA, USA) with four isolation channels was used to supply an output voltage range of 0.1 to 20 V_p-p_ with a frequency range of 0 to 16 MHz. The experiment was observed through an inverted microscope (Olympus IX 71, Olympus Corporation, Shinjuku-ku, Japan), and a fluorescent light source was used to excite the fluorescent nanocolloids. The experimental results were recorded in both video and photo formats using a high-speed charge-coupled device (CCD) camera (20 frames/s, Olympus DP 80, Olympus Corporation, Shinjuku-ku, Japan).

**Figure 1:**
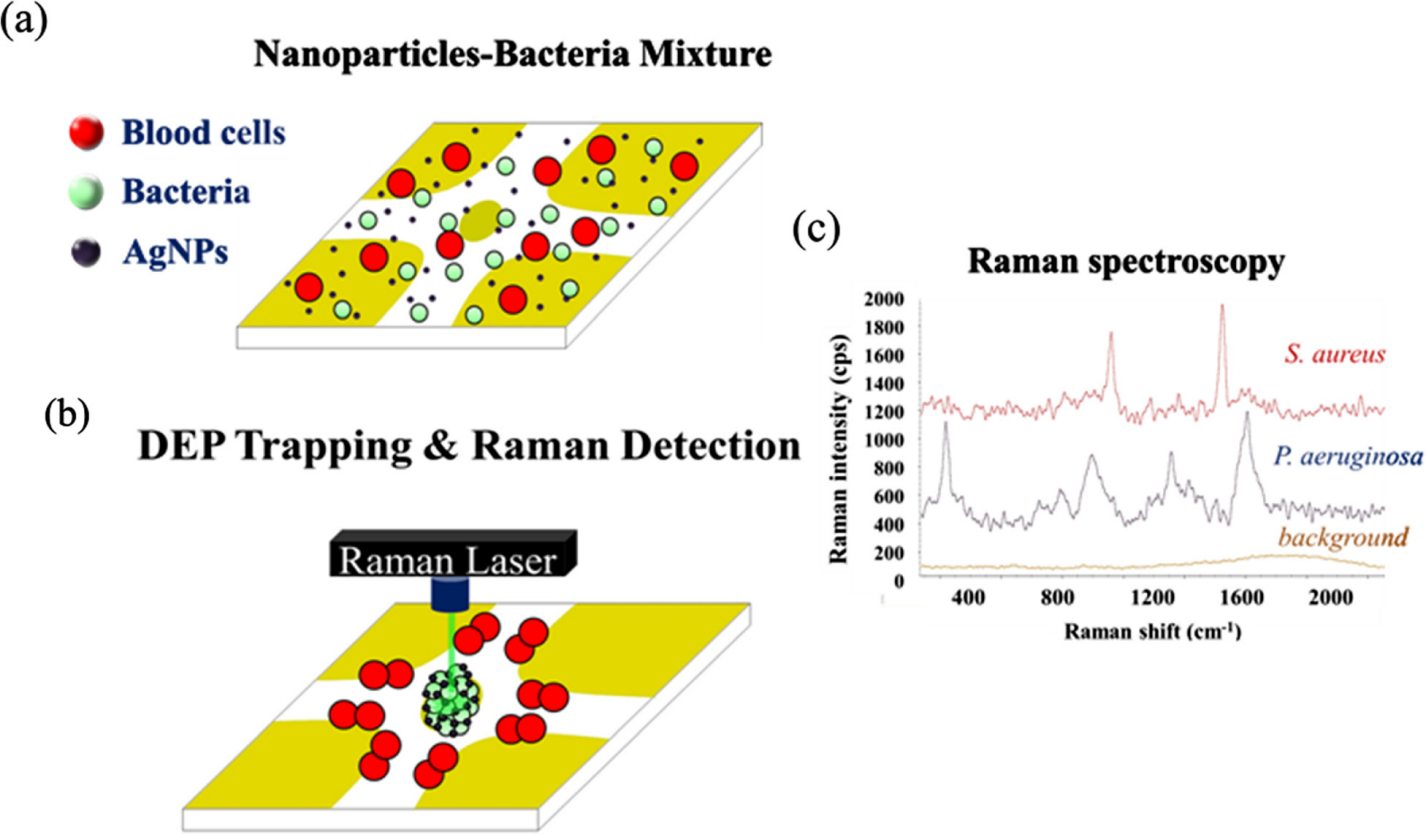
**Experimental flow chart. (a)** AgNPs were spiked and resuspended into the prepared bacteria solution. **(b)** AC voltage was applied to separate and collect the bacteria in the middle region. The AgNPs can also be trapped with the bacteria aggregate via the amplified positive DEP force. After bacteria-AgNP concentration and adsorption, the Raman laser was then irradiated to the bacteria-NP aggregate separated from the blood cells for the purpose of SERS identification. **(c)** On-chip identification of bacteria by comparing the detected SERS spectra to the spectra library.

An argon laser at 532 nm was used for excitation through an inverted microscope. The laser power at the sample position was around 1 mW, and the scattering light was collected using a 10× objective lens connected to a CCD. The Raman shift was calibrated using a signal of 520 cm^-1^ generated from a silicon wafer. All reported spectra of the exposure time were set to 5 s, and signal was accumulated two times in a range of 500 (approximately 2,000 cm^-1^). Rayleigh scattering was blocked using a holographic notch filter, and the tilted baselines of some SERS spectra were corrected to flat using OMNIC 8 software (Thermo Fisher Scientific, Waltham, MA, USA). The integrated experimental system is shown in Figure 1.

## Results and discussion

### Finite element simulation

**Figure 2:**
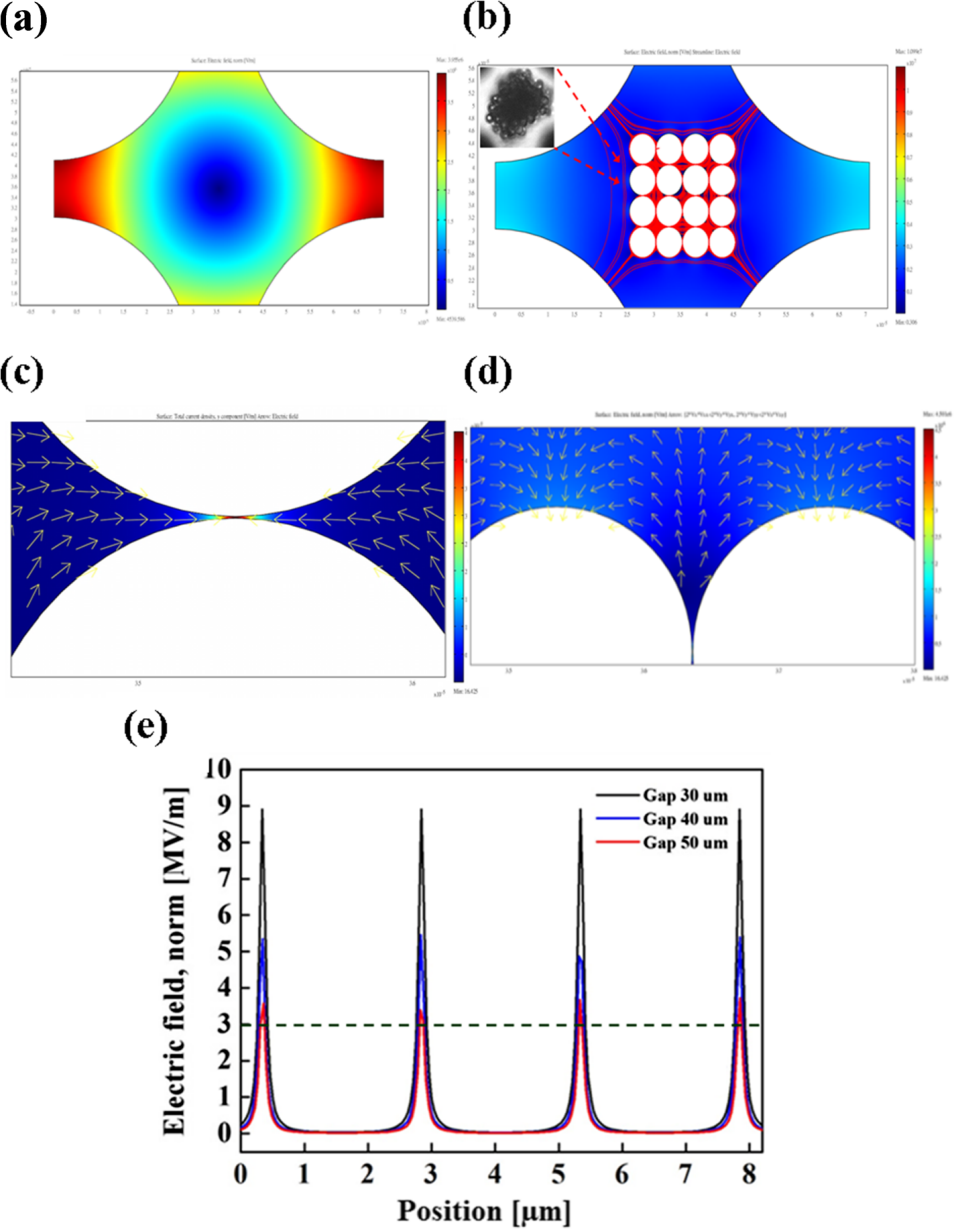
**Finite element simulation. (a)** The electric field distribution of a quadruple electrode. **(b)** The simulation result for the electric field distribution at the assembled microparticles. **(c)** After concentrating the microparticles, the applied electric field is focused and locally amplified at the assembled bead-bead gaps wherein an extremely high electric field is produced. The amplified electric field can induce a positive DEP for manipulating nanocolloids into the gaps of the assembled microparticles. **(d)** The simulation result indicates that the local surface of the assembled microparticles also generates a secondary high electric field region. **(e)** The strength of the amplified electric field generated from the different electrode gaps. The dashed line indicates the threshold strength of electric field for effectively manipulating several tens nanometers colloids.

Figure 2a,b shows the finite element simulation results for the electric field distribution without and with the microparticle assembly, respectively. The electric fields were solved numerically using finite element analysis software (Comsol Multiphysics 3.5, Comsol Ltd., Burlington, MA, USA). The electric scalar potential satisfies Poisson's equation, and the electric field and displacement are obtained from the electric potential gradient. The electric field distribution for the quadruple electrode shows that the high electric field regions exhibit at the electrode edges and that the center region between the four electrodes exhibits a lowest electric field region (Figure 2a). When the particles were induced with a negative DEP force, they were concentrated at the middle region to form a particle aggregate. Figure 2b (inset) shows a microscopic image of the DEP particle assembly. In Figure 2c, it can be seen that after concentrating the microparticles, the applied electric field is focused and locally amplified at the assembled bead-bead gaps such that the formed nanopores can produce an extremely high electric field for the purpose of manipulating the silver nanoparticles using a positive DEP force. The simulation results also demonstrate that the local surface of the assembled microparticles induces a secondary high electric field region in the tangential direction of the applied electric field, as shown in Figure 2d. This phenomenon could be attributed to the field-induced charge convection on the particle surface. The convected charges concentrate to the stagnation point, and thus, the high charge density generates a high electric field flux at that point [25]. Therefore, when the nanocolloids are induced with a positive DEP, they are not only effectively trapped into the bead-bead gaps but also trapped on the surfaces of the assembled particles by the amplified DEP force. In addition, in order to manipulate 20- to 50-nm particles, the electric field must be higher than 3 × 10^6^ V/m [26]. The better situation would be one in which the locally amplified electric field gradient is larger than the one produced by the electrode edges. Because the DEP force scales quadratically with respect to the electric field, the DEP force at the assembled microparticle is thus about 3 orders of magnitude higher than that generated by the planar electrodes and 1 order higher than that generated by the electrode edges, as shown in Figure 2e. Therefore, based on the required electric field strength, the electrode separation should be designed to be less than 50 μm, as shown in Figure 2e.

### Nanocolloid trapping mechanism using the dielectrophoretic microparticle assembly

**Figure 3:**
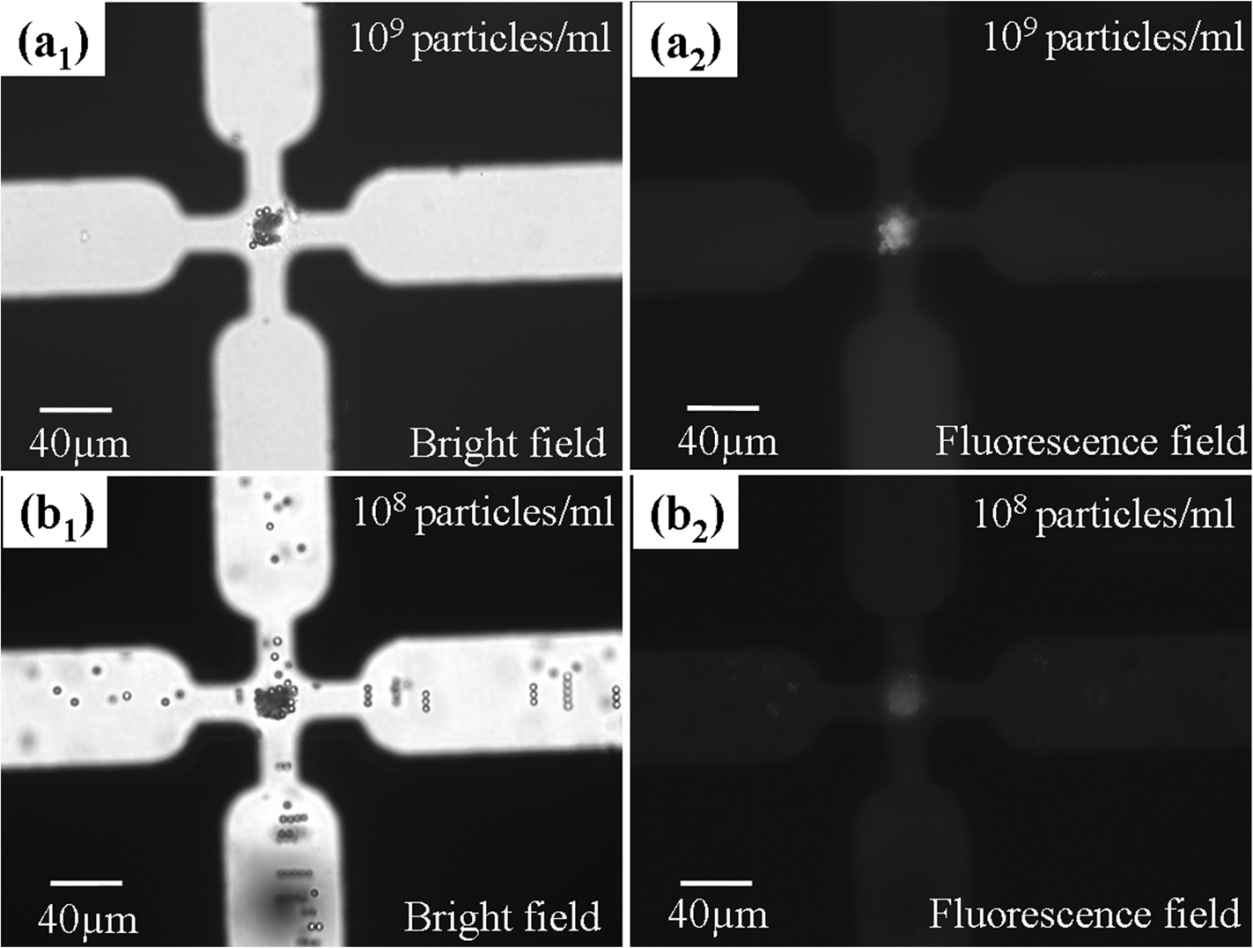
**Nanocolloid trapping mechanism. (a1)** Five micrometers was induced with a negative DEP force to be concentrated in the middle area. **(a2)** The DEP-assembled microparticle aggregate traps the fluorescent nanocolloids effectively, thus exhibiting an evident fluorescence phenomenon. **(b1, b2)** NanoDEP trapping result at a lower concentration of fluorescent nanocolloids.

Five-micrometer latex particles (no fluorescence) and 20-nm fluorescent nanocolloids were mixed and suspended in DI water with a concentration of 10^6^ and 10^9^ particles/ml, respectively. The previous study mentioned that nanoscale particles exhibit positive DEP at the frequency window of low frequency [27], and it has been shown that their cross-over frequency is with respect to the product of the Debye length and the particle size [26]. When an AC voltage of 15 V_p-p_ at a frequency of 100 kHz was supplied to the quadruple electrode, the negative DEP force caused 5 μm to be concentrated in the middle area of the weakest electric field region. At this frequency, the fluorescent nanocolloids were induced with a positive DEP force that manipulated the fluorescent nanocolloids into the microparticle aggregate. After applying voltage for 3 min, we switched the observation from a bright field to a fluorescent field. The result clearly showed that the DEP-formed microparticle aggregate exhibits an evident fluorescence phenomenon, as shown in Figure 3a,b. This process can be utilized to validate and illustrate that the fluorescent nanocolloids were effectively trapped into the bead-bead gaps of the assembled microparticles due to the amplified positive DEP force and also were trapped on the local surface of the microparticles. Figure 3b shows the nanoDEP trapping result under the same condition but at a lower concentration of fluorescent nanocolloids.

### Optimal conditions and on-chip SERS identification of bacteria

**Figure 4:**
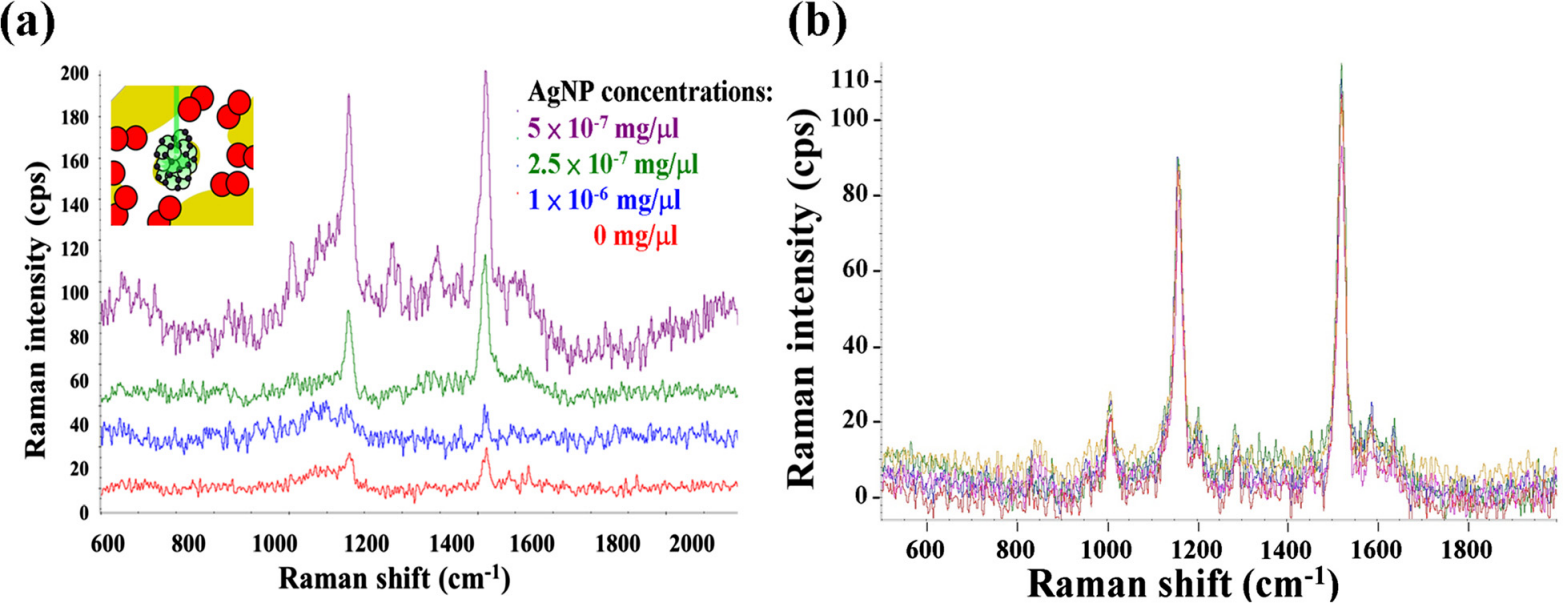
**Bacteria Raman signals and spectra of *S*. *aureus*. (a)** The bacteria solution with different AgNP concentrations of 2.5 × 10^-7^, 5 × 10^-7^, and 1 × 10^-6^ mg/μl was adjusted to investigate the optimal AgNP condition for SERS resulting in an optimal AgNP concentration being found at 5 × 10^-7^ mg/μl. **(b)** Spectra of *S. aureus* that were detected via the amplified DEP AgNP-enhanced Raman five runs using five different chips.

The bacteria (*S. aureus*) was found to exhibit strong positive DEP (pDEP) at frequencies above 3 MHz and strong negative DEP (nDEP) below 2 MHz, while blood cells exhibited strong nDEP at frequencies below 500 kHz and strong pDEP behavior above 800 kHz. AgNPs were spiked into the prepared bacteria solution to adjust to a constant bacteria concentration of 10^7^ CFU/ml with different AgNP concentrations. At frequencies below 2 MHz, all bacteria exhibited nDEP in the conductive medium with a conductivity of 1 mS/cm and were trapped in the middle of the electrode gap. Metal-based nanocolloids have been shown to exhibit a high positive DEP force at both low and high frequencies due to their high conductivity and polarizability [28]. Therefore, a voltage of 15 V_p-p_ at a frequency of 1 MHz was applied to simultaneously concentrate the bacteria using negative DEP and to trap the AgNPs by the bacteria assembly that produced the amplified positive DEP force. To investigate the optimal AgNP concentration in the bacteria solution for the enhancement of the Raman signal, the different AgNP concentrations of 2.5 × 10^-7^, 5 × 10^-7^, and 1 × 10^-6^ mg/μl were adjusted. Figure 4a shows the bacteria Raman signals that were measured after DEP concentration for 3 min at the different AgNP concentrations. The optimal AgNP concentration was found at 5 × 10^-7^ mg/μl. Under this condition, the SERS intensity was at least 5-fold higher than that of the normal Raman spectrum measured from the bacteria sample without AgNP spiking, which was proof of the effectiveness of the concept for the DEP-assisted NP-bacteria adsorption intended to enhance the Raman signal. The minimal gap for assembled microparticles has been calculated to be roughly 10 nm (approximately 2*λ*, *λ* is the thickness of the double layer) at a conductivity of 1 mS/cm [9]; thus, the electric field is compressed, and the DEP force is locally amplified at the assembled bead-bead gaps such that the nanostructures produce an extremely high positive DEP force for manipulating AgNPs/nanocolloids, as shown in Figure 2a. Another assisted mechanism for AgNP-bacteria adsorption could be attributed to the electric field-induced dipole-dipole interaction [29,30]. Figure 4b shows five spectra of *S. aureus* that were detected for five times by five different chips. This result demonstrates good spectral reproducibility via dielectrophoresistic-assisted AgNP-bacteria sorption.

**Figure 5:**
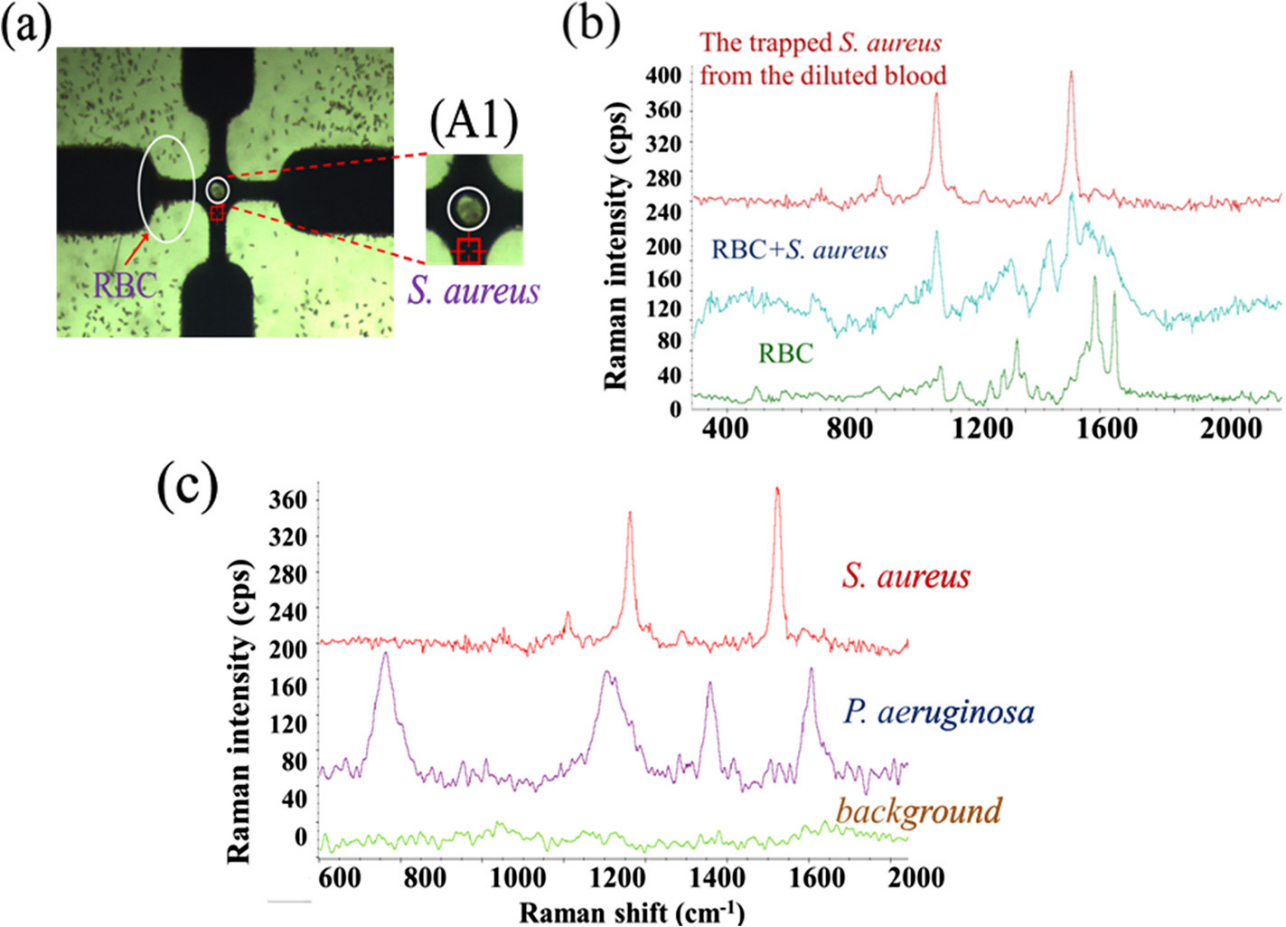
**Separation and concentration of bacteria, SERS spectra, and detection result. (a)** Separation and concentration of bacteria from a BC-bacteria mixture. Inset A1 shows a higher magnification photo of the center area; there is a high density of bacteria aggregate without blood cells at the center. **(b)** The SERS spectra of RBC, RBC-bacteria mixture, and the *S. aureus* dielectrokinetically separated from blood. **(c)** The detection result shows very distinct fingerprints of *S. aureus* and *P. aeruginosa* that were measured after separation and AgNP-bacteria sorption.

The blood cell-bacteria mixture was also used to demonstrate that our platform is capable of identifying bacteria from a diluted blood sample. Therefore, the DEP approach was also used to separate bacteria and blood cells. A voltage of 15 V_p-p_ at a frequency of 1 MHz was applied to separate the bacteria and blood cells based on their different DEP behaviors. Under this electrical condition, the blood cells were attracted to the electrode edges by the positive DEP force, while the bacteria experienced a negative DEP force and were trapped and concentrated in the middle region between the quadruple electrodes where there is a high density of bacteria aggregate to be Raman-detected, as shown in Figure 5a and inset A1. After bacteria separation and concentration, the trapped bacteria aggregate continued to experience the amplified DEP force in order to adsorb the AgNPs into the bacteria aggregate for 3 min. The Raman laser spot was then irradiated to the bacteria-NP aggregate separated from the blood cells for the purpose of SERS identification of the concentrated bacteria. The red and green lines in Figure 5b indicate the Raman spectra of the red blood cell (RBC) and RBC-bacteria mixture, respectively. The results demonstrate that the spectrum of the RBC-bacteria mixture is very similar to that of the RBC signature. This makes it difficult to identify the target bacteria using the Raman technique without a separation procedure. On the other hand, a pure SERS signature of bacteria was obtained by directing a laser spot at the bacteria aggregate separated from the blood cells after applying a predetermined separation and trapping condition. Figure 5c shows very distinct fingerprints of *S. aureus* and *P. aeruginosa* that were measured after separation and AgNP-bacteria sorption from a bacteria-blood mixture. The background was measured from the diluted human blood without any bacteria after electrokinetically trapping both the blood cells and bacteria on the electrode edges at a frequency of 5 MHz. The results show that this technique can be used to trap bacteria from a sample containing blood cells, that its Raman signal can be enhanced via AgNP-bacteria sorption to determine the presence of blood infections, and that it can carry out on-chip identification of bacteria in bacteremia by comparing the detected SERS spectra to the spectra library. This method offers a number of potential advantages over conventional methods for cell/bacteria/virus identification, including extremely rapid speed, low cost for each detection, and simple process requirements.

## Conclusions

A novel mechanism for dielectrophoretic trapping of nanoscale particles through the use of a microparticle assembly was demonstrated for the purpose of effectively trapping nanocolloids using the amplified positive DEP force. The amplified electric field is shown to be 2 orders higher than the original middle region, and thus, the DEP force at these local regions can be predicted as 4 orders higher. The appropriate design for this trapping mechanism is one in which the gaps of quadruple electrodes are smaller than 50 μm in order to achieve a sufficient electric field strength needed for manipulating nanocolloids using the amplified positive DEP force. This mechanism was also used for SERS identification of bacteria from diluted blood successfully. The bacteria and blood cells were separated employing their different DEP behaviors, and furthermore, the concentrated bacteria produced an amplified positive DEP force for adsorption of AgNPs on the bacteria surface. The enhancement of SERS was at least 5-fold higher at an optimal AgNP concentration of 5 × 10^-7^ mg/μl when compared with the normal Raman spectrum. These results demonstrate good spectral reproducibility via dielectrophoresis-assisted AgNP-bacteria sorption. This technique could be readily used for the rapid detection of pathogens in human blood after blood culturing for approximately 12 h. Compared to the current method in the hospital, after blood culturing, this simple and rapid platform could accelerate the detection rate from 2 days to a few minutes. In the future, this approach could be widely used for bead-based hybridization and immunoassays.

## Competing interests

The authors declare that they have no competing interests.

## Authors' contributions

I-FC conceived and designed the experiments. R-JL and T-YC performed the DEP and Raman/SERS experiments, respectively. I-FC and H-WW wrote the paper and supervised this study. All authors read and approved the final manuscript.
